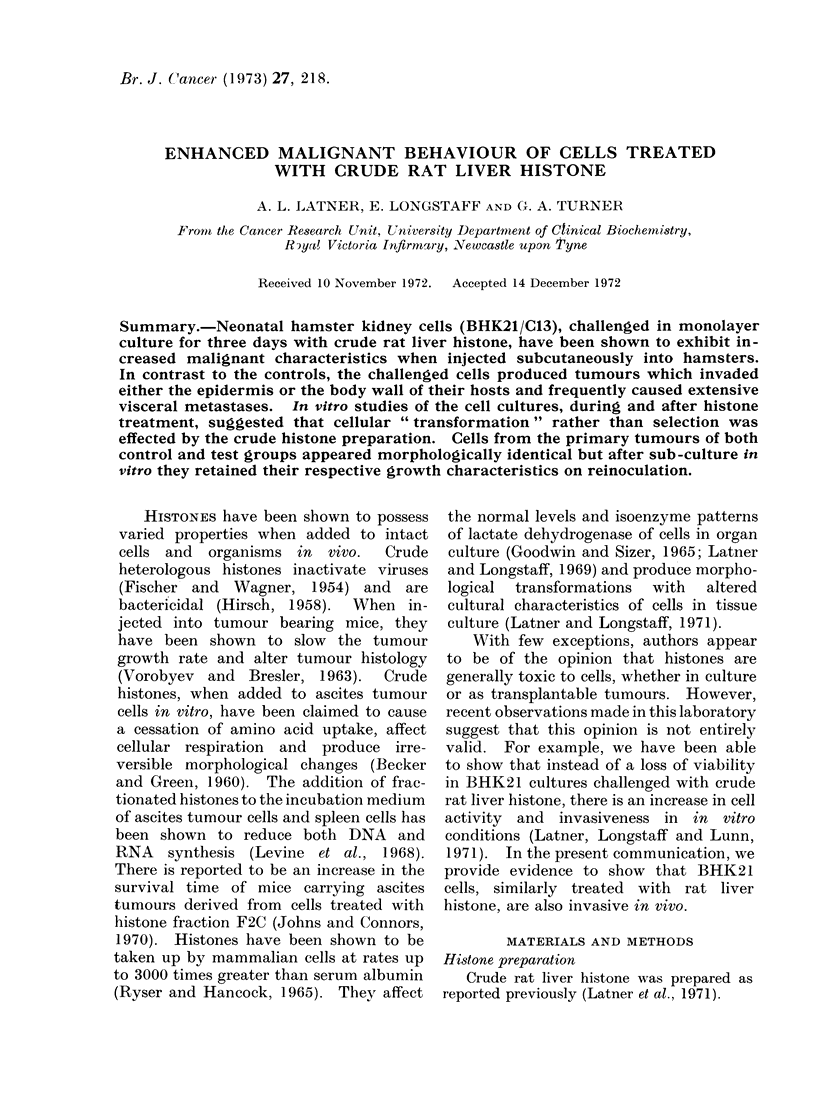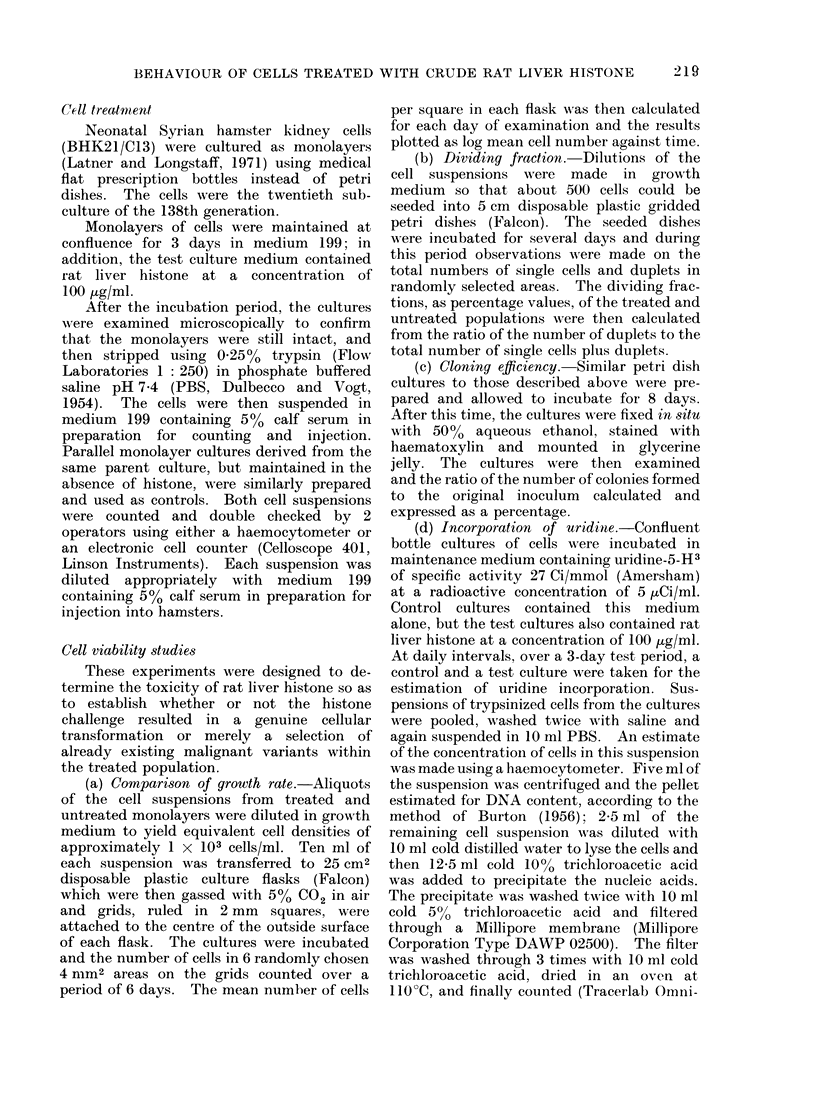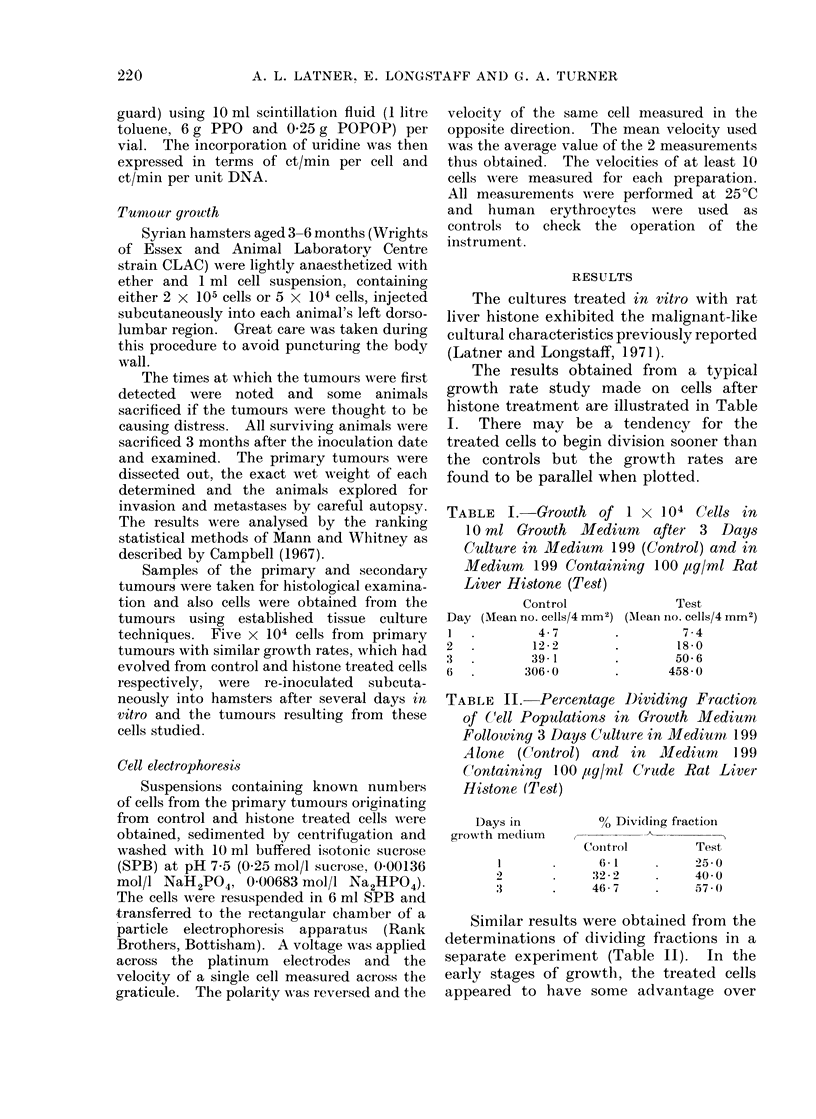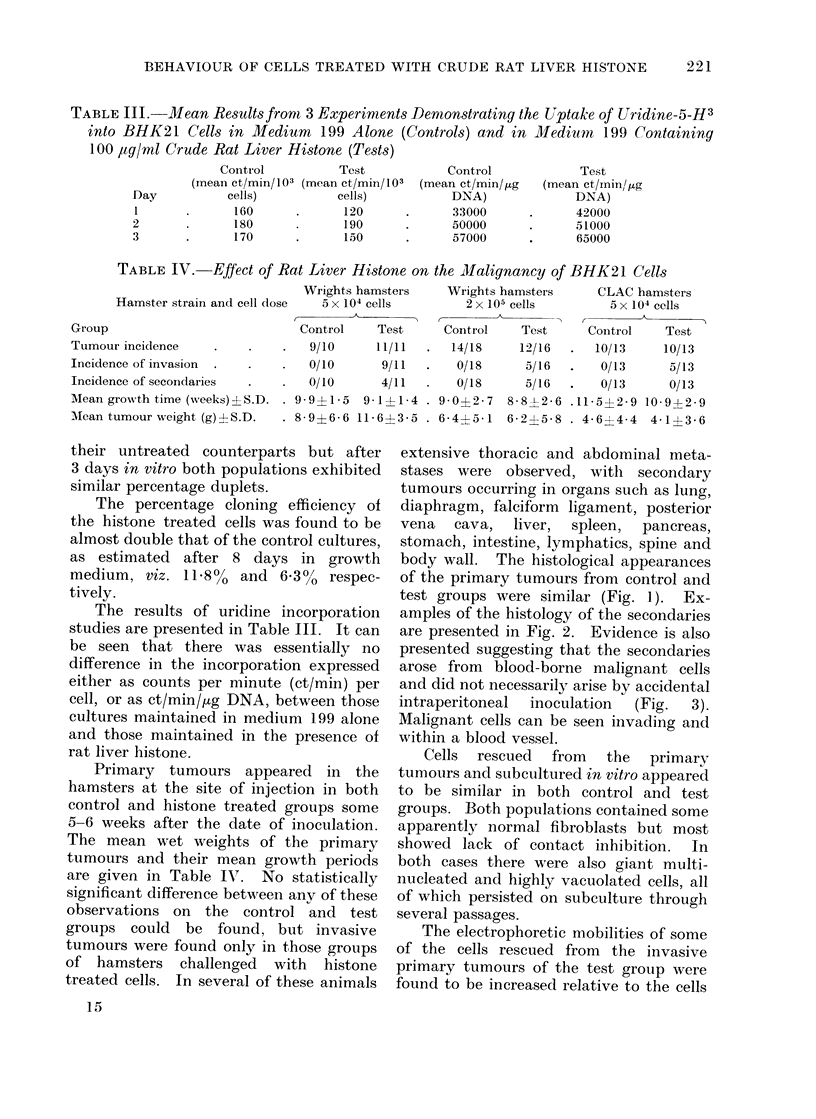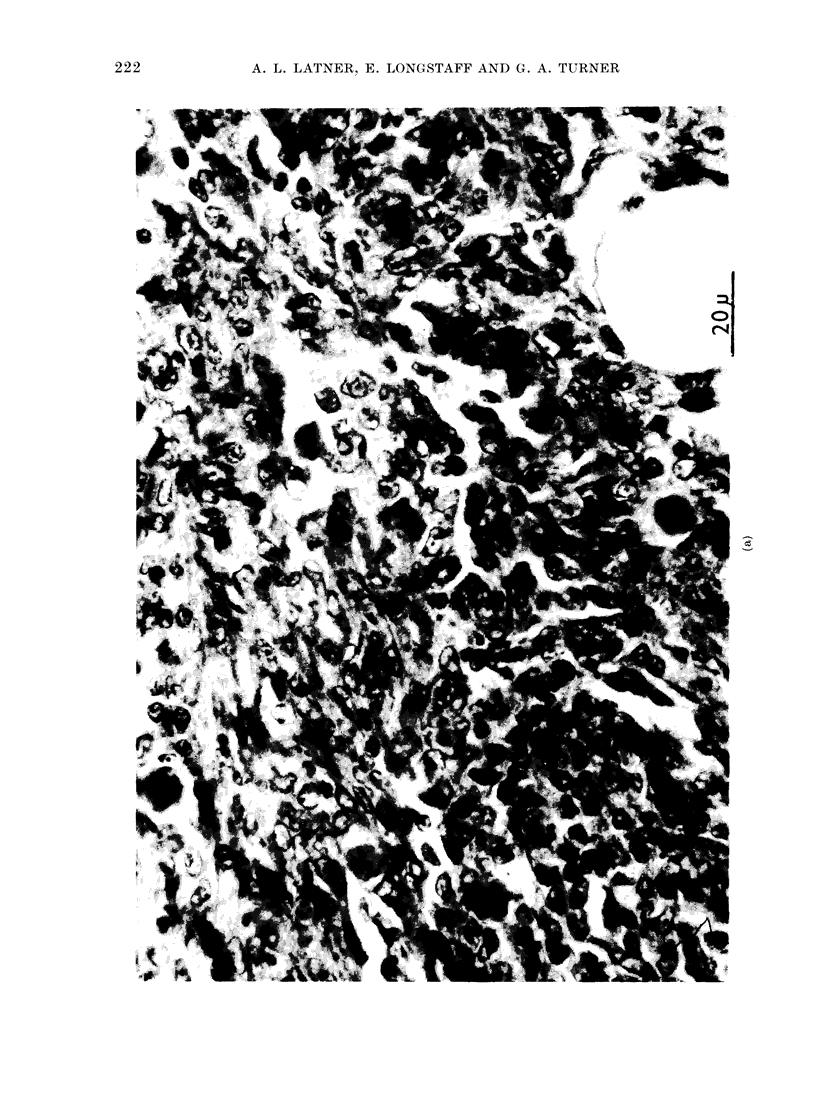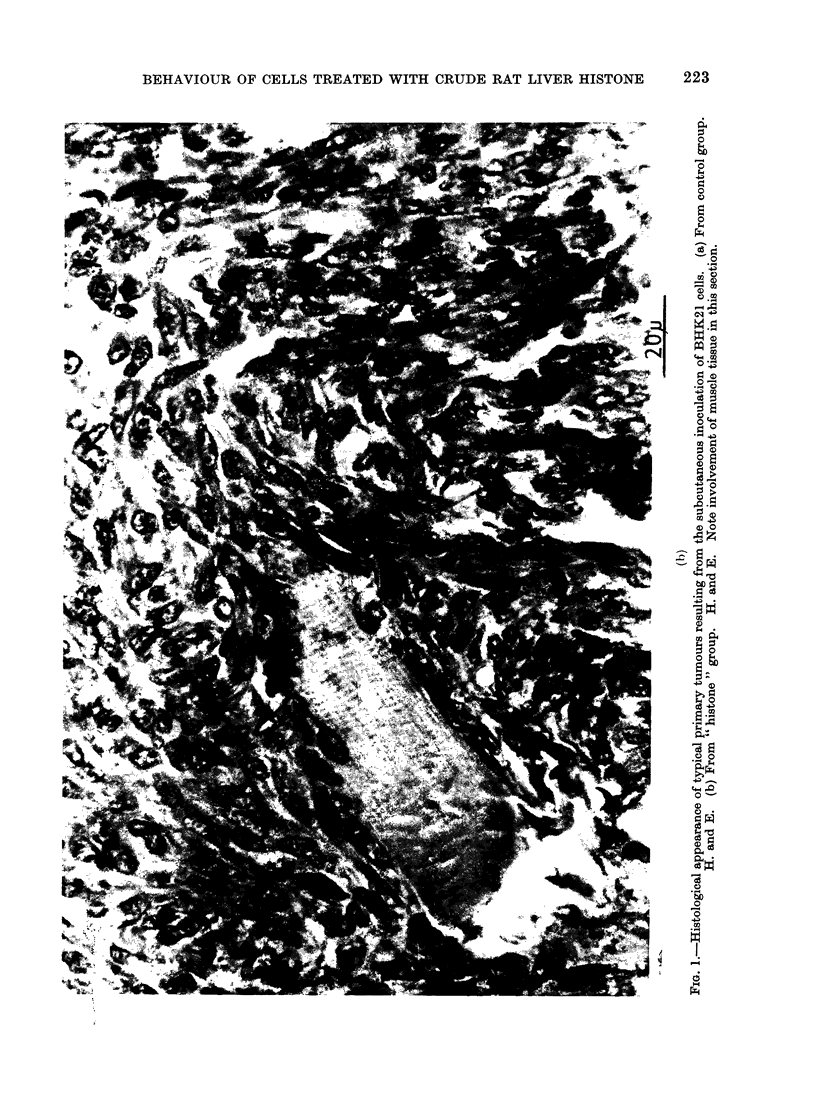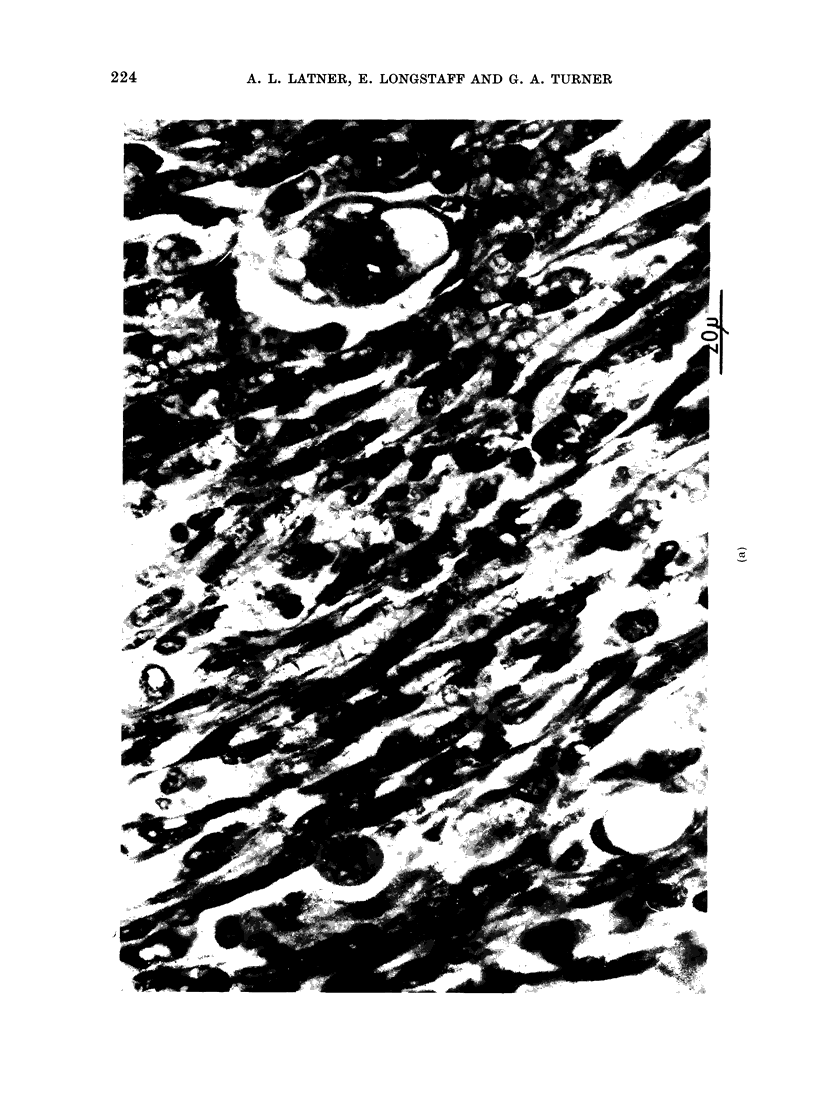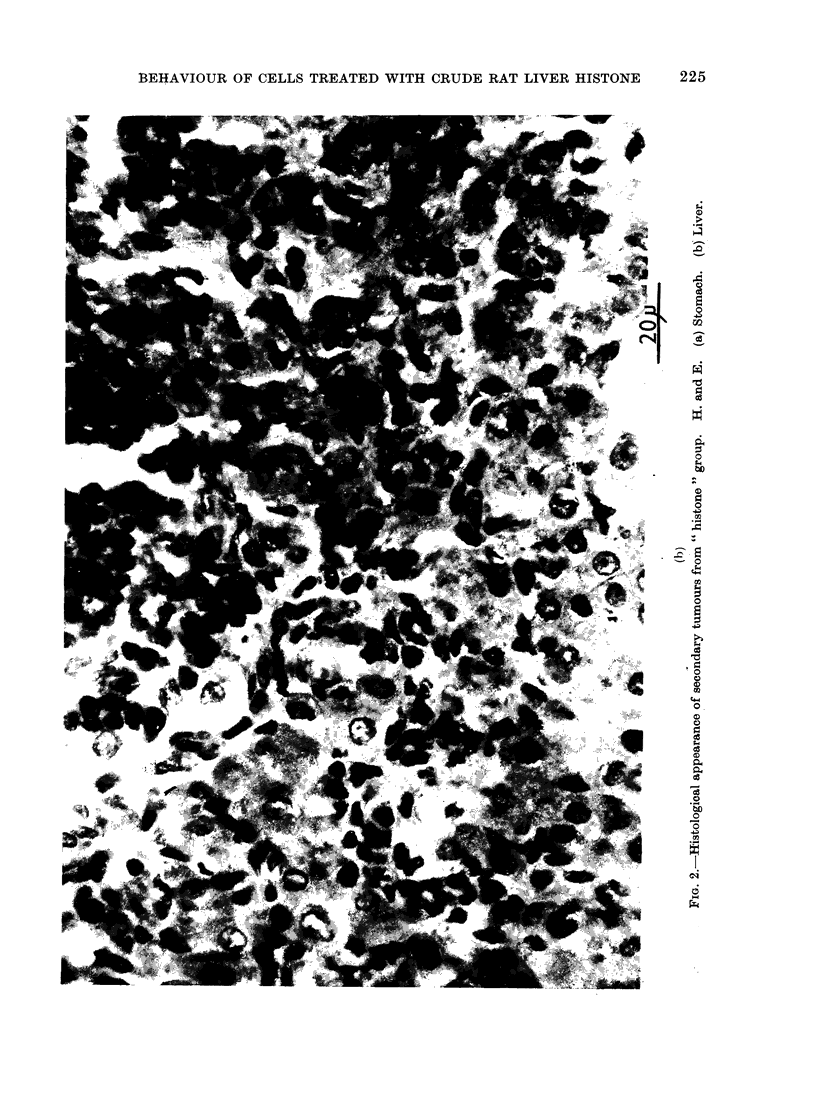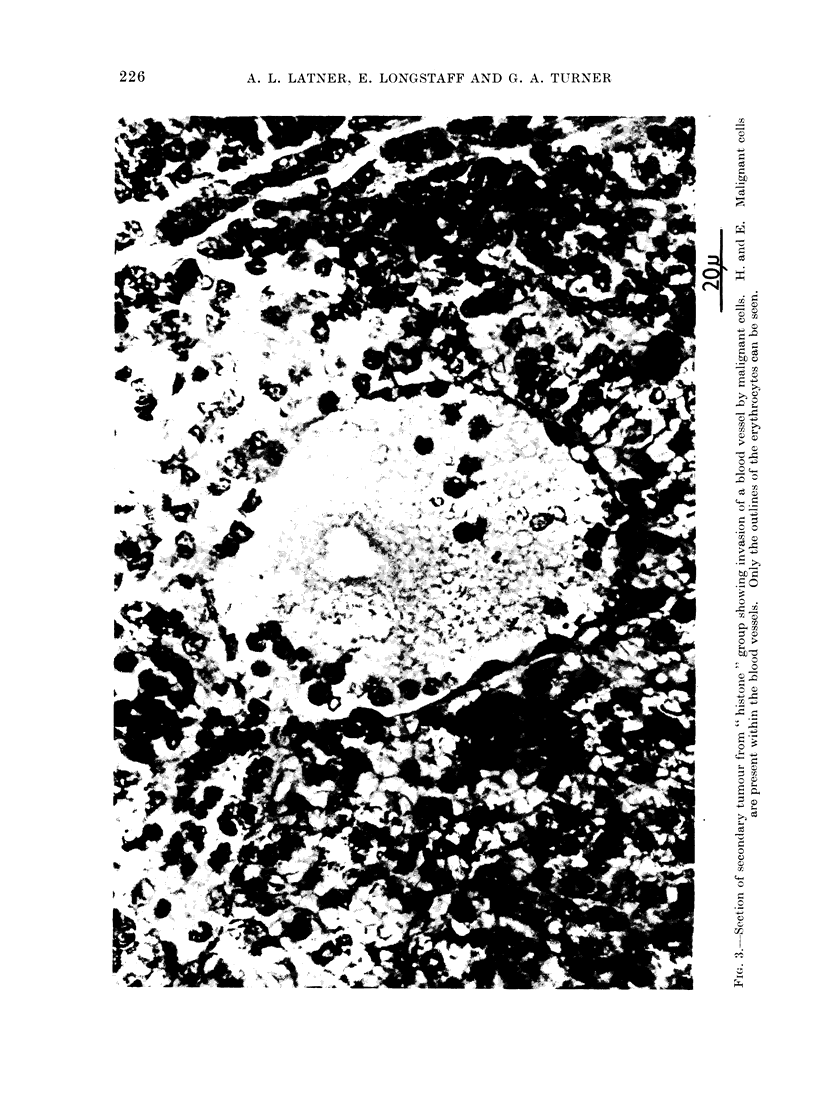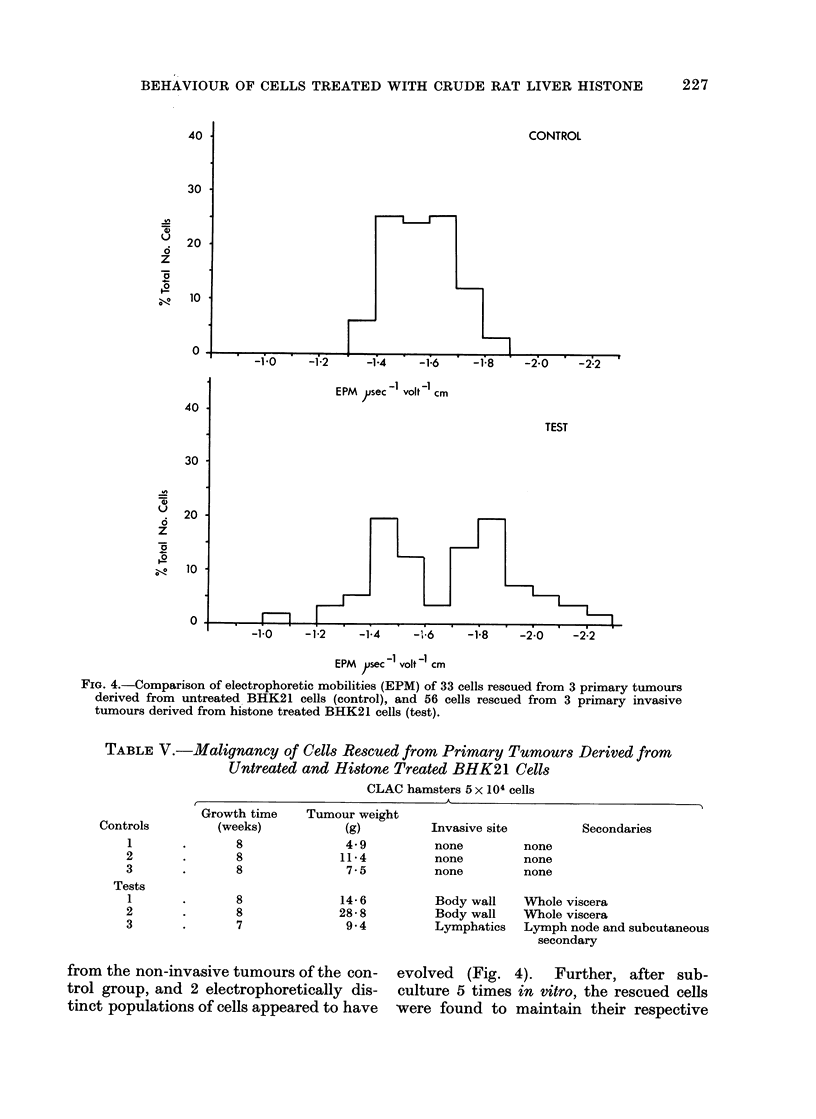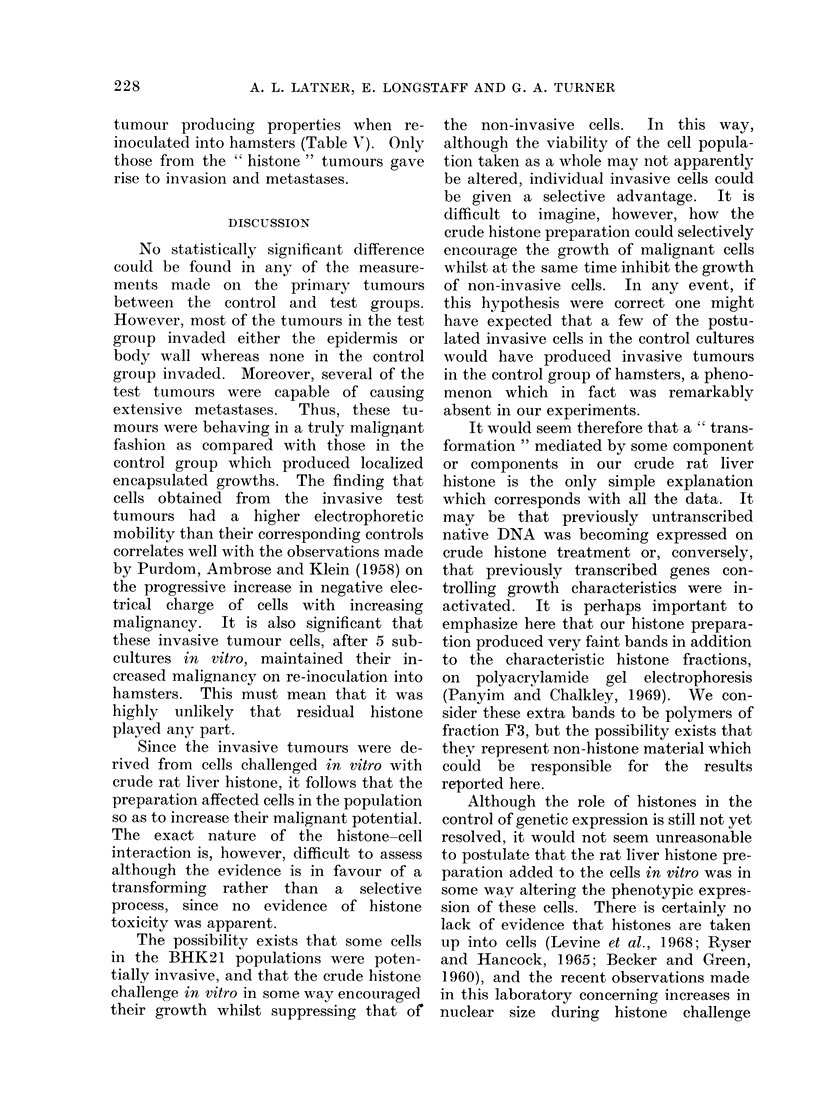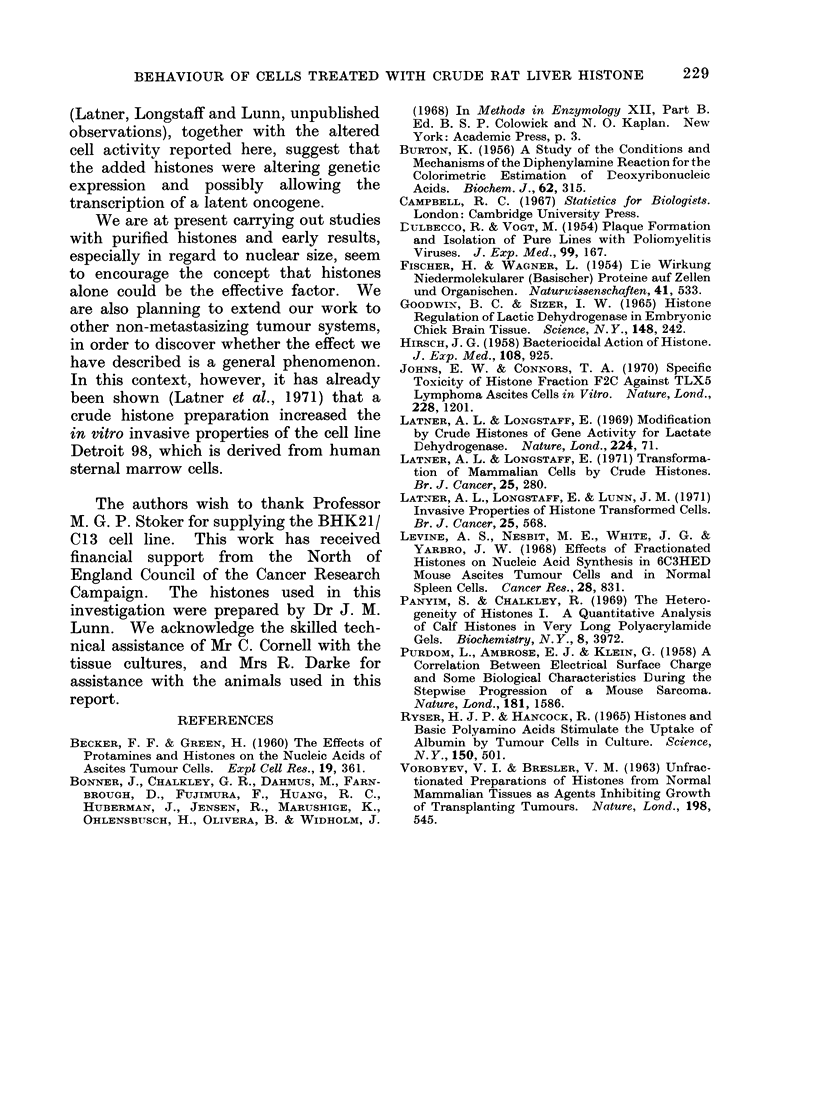# Enhanced Malignant Behaviour of Cells Treated with Crude Rat Liver Histone

**DOI:** 10.1038/bjc.1973.27

**Published:** 1973-03

**Authors:** A. L. Latner, E. Longstaff, G. A. Turner

## Abstract

**Images:**


*Br. J. Cancer* (1973) **27**, 218.

### ENHANCED MALIGNANT BEHAVIOUR OF CELLS TREATED WITH CRUDE RAT LIVER HISTONE

A. L. LATNER, E. LONGSTAFF AND G. A. TURNER

*From the Cancer Research Unit, University Department of Clinical Biochemistry, Royal Victoria Infirmary, Newcastle upon Tyne*

Received 10 November 1972. Accepted 14 December 1972

**Summary.—Neonatal hamster kidney cells (BHK21/C13), challenged in monolayer culture for three days with crude rat liver histone, have been shown to exhibit increased malignant characteristics when injected subcutaneously into hamsters. In contrast to the controls, the challenged cells produced tumours which invaded either the epidermis or the body wall of their hosts and frequently caused extensive visceral metastases. *In vitro* studies of the cell cultures, during and after histone treatment, suggested that cellular "transformation" rather than selection was effected by the crude histone preparation. Cells from the primary tumours of both control and test groups appeared morphologically identical but after sub-culture *in vitro* they retained their respective growth characteristics on reinoculation.**

HISTONES have been shown to possess varied properties when added to intact cells and organisms *in vivo*. Crude heterologous histones inactivate viruses (Fischer and Wagner, 1954) and are bactericidal (Hirsch, 1958). When injected into tumour bearing mice, they have been shown to slow the tumour growth rate and alter tumour histology (Vorobyev and Bresler, 1963). Crude histones, when added to ascites tumour cells *in vitro*, have been claimed to cause a cessation of amino acid uptake, affect cellular respiration and produce irreversible morphological changes (Becker and Green, 1960). The addition of fractionated histones to the incubation medium of ascites tumour cells and spleen cells has been shown to reduce both DNA and RNA synthesis (Levine *et al.*, 1968). There is reported to be an increase in the survival time of mice carrying ascites tumours derived from cells treated with histone fraction F2C (Johns and Connors, 1970). Histones have been shown to be taken up by mammalian cells at rates up to 3000 times greater than serum albumin (Ryser and Hancock, 1965). They affect the normal levels and isoenzyme patterns of lactate dehydrogenase of cells in organ culture (Goodwin and Sizer, 1965; Latner and Longstaff, 1969) and produce morphological transformations with altered cultural characteristics of cells in tissue culture (Latner and Longstaff, 1971).

With few exceptions, authors appear to be of the opinion that histones are generally toxic to cells, whether in culture or as transplantable tumours. However, recent observations made in this laboratory suggest that this opinion is not entirely valid. For example, we have been able to show that instead of a loss of viability in BHK21 cultures challenged with crude rat liver histone, there is an increase in cell activity and invasiveness in *in vitro* conditions (Latner, Longstaff and Lunn, 1971). In the present communication, we provide evidence to show that BHK21 cells, similarly treated with rat liver histone, are also invasive *in vivo*.

### MATERIALS AND METHODS

### *Histone preparation*

Crude rat liver histone was prepared as reported previously (Latner *et al.*, 1971).

*Cell treatment*

Neonatal Syrian hamster kidney cells (BHK21/C13) were cultured as monolayers (Latner and Longstaff, 1971) using medical flat prescription bottles instead of petri dishes. The cells were the twentieth subculture of the 138th generation.

Monolayers of cells were maintained at confluence for 3 days in medium 199; in addition, the test culture medium contained rat liver histone at a concentration of 100 $\mu$g/ml.

After the incubation period, the cultures were examined microscopically to confirm that the monolayers were still intact, and then stripped using 0·25% trypsin (Flow Laboratories 1 : 250) in phosphate buffered saline pH 7·4 (PBS, Dulbecco and Vogt, 1954). The cells were then suspended in medium 199 containing 5% calf serum in preparation for counting and injection. Parallel monolayer cultures derived from the same parent culture, but maintained in the absence of histone, were similarly prepared and used as controls. Both cell suspensions were counted and double checked by 2 operators using either a haemocytometer or an electronic cell counter (Celloscope 401, Linson Instruments). Each suspension was diluted appropriately with medium 199 containing 5% calf serum in preparation for injection into hamsters.

*Cell viability studies*

These experiments were designed to determine the toxicity of rat liver histone so as to establish whether or not the histone challenge resulted in a genuine cellular transformation or merely a selection of already existing malignant variants within the treated population.

(a) *Comparison of growth rate.*—Aliquots of the cell suspensions from treated and untreated monolayers were diluted in growth medium to yield equivalent cell densities of approximately $1 \times 10^3$ cells/ml. Ten ml of each suspension was transferred to 25 cm$^2$ disposable plastic culture flasks (Falcon) which were then gassed with 5% $CO_2$ in air and grids, ruled in 2 mm squares, were attached to the centre of the outside surface of each flask. The cultures were incubated and the number of cells in 6 randomly chosen 4 mm$^2$ areas on the grids counted over a period of 6 days. The mean number of cells per square in each flask was then calculated for each day of examination and the results plotted as log mean cell number against time.

(b) *Dividing fraction.*—Dilutions of the cell suspensions were made in growth medium so that about 500 cells could be seeded into 5 cm disposable plastic gridded petri dishes (Falcon). The seeded dishes were incubated for several days and during this period observations were made on the total numbers of single cells and duplets in randomly selected areas. The dividing fractions, as percentage values, of the treated and untreated populations were then calculated from the ratio of the number of duplets to the total number of single cells plus duplets.

(c) *Cloning efficiency.*—Similar petri dish cultures to those described above were prepared and allowed to incubate for 8 days. After this time, the cultures were fixed *in situ* with 50% aqueous ethanol, stained with haematoxylin and mounted in glycerine jelly. The cultures were then examined and the ratio of the number of colonies formed to the original inoculum calculated and expressed as a percentage.

(d) *Incorporation of uridine.*—Confluent bottle cultures of cells were incubated in maintenance medium containing uridine-5-$H^3$ of specific activity 27 Ci/mmol (Amersham) at a radioactive concentration of 5 $\mu$Ci/ml. Control cultures contained this medium alone, but the test cultures also contained rat liver histone at a concentration of 100 $\mu$g/ml. At daily intervals, over a 3-day test period, a control and a test culture were taken for the estimation of uridine incorporation. Suspensions of trypsinized cells from the cultures were pooled, washed twice with saline and again suspended in 10 ml PBS. An estimate of the concentration of cells in this suspension was made using a haemocytometer. Five ml of the suspension was centrifuged and the pellet estimated for DNA content, according to the method of Burton (1956); 2·5 ml of the remaining cell suspension was diluted with 10 ml cold distilled water to lyse the cells and then 12·5 ml cold 10% trichloroacetic acid was added to precipitate the nucleic acids. The precipitate was washed twice with 10 ml cold 5% trichloroacetic acid and filtered through a Millipore membrane (Millipore Corporation Type DAWP 02500). The filter was washed through 3 times with 10 ml cold trichloroacetic acid, dried in an oven at 110°C, and finally counted (Tracerlab Omniguard) using 10 ml scintillation fluid (1 litre toluene, 6 g PPO and 0·25 g POPOP) per vial. The incorporation of uridine was then expressed in terms of ct/min per cell and ct/min per unit DNA.

*Tumour growth*

Syrian hamsters aged 3–6 months (Wrights of Essex and Animal Laboratory Centre strain CLAC) were lightly anaesthetized with ether and 1 ml cell suspension, containing either $2 \times 10^5$ cells or $5 \times 10^4$ cells, injected subcutaneously into each animal's left dorsolumbar region. Great care was taken during this procedure to avoid puncturing the body wall.

The times at which the tumours were first detected were noted and some animals sacrificed if the tumours were thought to be causing distress. All surviving animals were sacrificed 3 months after the inoculation date and examined. The primary tumours were dissected out, the exact wet weight of each determined and the animals explored for invasion and metastases by careful autopsy. The results were analysed by the ranking statistical methods of Mann and Whitney as described by Campbell (1967).

Samples of the primary and secondary tumours were taken for histological examination and also cells were obtained from the tumours using established tissue culture techniques. Five $\times 10^4$ cells from primary tumours with similar growth rates, which had evolved from control and histone treated cells respectively, were re-inoculated subcutaneously into hamsters after several days *in vitro* and the tumours resulting from these cells studied.

*Cell electrophoresis*

Suspensions containing known numbers of cells from the primary tumours originating from control and histone treated cells were obtained, sedimented by centrifugation and washed with 10 ml buffered isotonic sucrose (SPB) at pH 7·5 (0·25 mol/l sucrose, 0·00136 mol/l $NaH_2PO_4$, 0·00683 mol/l $Na_2HPO_4$). The cells were resuspended in 6 ml SPB and transferred to the rectangular chamber of a particle electrophoresis apparatus (Rank Brothers, Bottisham). A voltage was applied across the platinum electrodes and the velocity of a single cell measured across the graticule. The polarity was reversed and the velocity of the same cell measured in the opposite direction. The mean velocity used was the average value of the 2 measurements thus obtained. The velocities of at least 10 cells were measured for each preparation. All measurements were performed at 25°C and human erythrocytes were used as controls to check the operation of the instrument.

RESULTS

The cultures treated *in vitro* with rat liver histone exhibited the malignant-like cultural characteristics previously reported (Latner and Longstaff, 1971).

The results obtained from a typical growth rate study made on cells after histone treatment are illustrated in Table I. There may be a tendency for the treated cells to begin division sooner than the controls but the growth rates are found to be parallel when plotted.

TABLE I.—*Growth of* $1 \times 10^4$ *Cells in* 10 *ml Growth Medium after* 3 *Days Culture in Medium* 199 (*Control*) *and in Medium* 199 *Containing* 100 *μg/ml Rat Liver Histone* (*Test*)

| Day | Control (Mean no. cells/4 mm²) | Test (Mean no. cells/4 mm²) |
|---|---|---|
| 1 | 4·7 | 7·4 |
| 2 | 12·2 | 18·0 |
| 3 | 39·1 | 50·6 |
| 6 | 306·0 | 458·0 |

TABLE II.—*Percentage Dividing Fraction of Cell Populations in Growth Medium Following* 3 *Days Culture in Medium* 199 *Alone* (*Control*) *and in Medium* 199 *Containing* 100 *μg/ml Crude Rat Liver Histone* (*Test*)

| Days in growth medium | % Dividing fraction | |
|---|---|---|
| | Control | Test |
| 1 | 6·1 | 25·0 |
| 2 | 32·2 | 40·0 |
| 3 | 46·7 | 57·0 |

Similar results were obtained from the determinations of dividing fractions in a separate experiment (Table II). In the early stages of growth, the treated cells appeared to have some advantage over

TABLE III.—*Mean Results from 3 Experiments Demonstrating the Uptake of Uridine-5-$H^3$ into BHK21 Cells in Medium 199 Alone (Controls) and in Medium 199 Containing 100 μg/ml Crude Rat Liver Histone (Tests)*

| Day | Control (mean ct/min/$10^3$ cells) | Test (mean ct/min/$10^3$ cells) | Control (mean ct/min/μg DNA) | Test (mean ct/min/μg DNA) |
|---|---|---|---|---|
| 1 | 160 | 120 | 33000 | 42000 |
| 2 | 180 | 190 | 50000 | 51000 |
| 3 | 170 | 150 | 57000 | 65000 |

TABLE IV.—*Effect of Rat Liver Histone on the Malignancy of BHK21 Cells*

| Hamster strain and cell dose | Wrights hamsters $5 \times 10^4$ cells | | Wrights hamsters $2 \times 10^5$ cells | | CLAC hamsters $5 \times 10^4$ cells | |
|---|---|---|---|---|---|---|
| Group | Control | Test | Control | Test | Control | Test |
| Tumour incidence | 9/10 | 11/11 | 14/18 | 12/16 | 10/13 | 10/13 |
| Incidence of invasion | 0/10 | 9/11 | 0/18 | 5/16 | 0/13 | 5/13 |
| Incidence of secondaries | 0/10 | 4/11 | 0/18 | 5/16 | 0/13 | 0/13 |
| Mean growth time (weeks) ± S.D. | 9·9 ± 1·5 | 9·1 ± 1·4 | 9·0 ± 2·7 | 8·8 ± 2·6 | 11·5 ± 2·9 | 10·9 ± 2·9 |
| Mean tumour weight (g) ± S.D. | 8·9 ± 6·6 | 11·6 ± 3·5 | 6·4 ± 5·1 | 6·2 ± 5·8 | 4·6 ± 4·4 | 4·1 ± 3·6 |

their untreated counterparts but after 3 days *in vitro* both populations exhibited similar percentage duplets.

The percentage cloning efficiency of the histone treated cells was found to be almost double that of the control cultures, as estimated after 8 days in growth medium, *viz.* 11·8% and 6·3% respectively.

The results of uridine incorporation studies are presented in Table III. It can be seen that there was essentially no difference in the incorporation expressed either as counts per minute (ct/min) per cell, or as ct/min/μg DNA, between those cultures maintained in medium 199 alone and those maintained in the presence of rat liver histone.

Primary tumours appeared in the hamsters at the site of injection in both control and histone treated groups some 5–6 weeks after the date of inoculation. The mean wet weights of the primary tumours and their mean growth periods are given in Table IV. No statistically significant difference between any of these observations on the control and test groups could be found, but invasive tumours were found only in those groups of hamsters challenged with histone treated cells. In several of these animals extensive thoracic and abdominal metastases were observed, with secondary tumours occurring in organs such as lung, diaphragm, falciform ligament, posterior vena cava, liver, spleen, pancreas, stomach, intestine, lymphatics, spine and body wall. The histological appearances of the primary tumours from control and test groups were similar (Fig. 1). Examples of the histology of the secondaries are presented in Fig. 2. Evidence is also presented suggesting that the secondaries arose from blood-borne malignant cells and did not necessarily arise by accidental intraperitoneal inoculation (Fig. 3). Malignant cells can be seen invading and within a blood vessel.

Cells rescued from the primary tumours and subcultured *in vitro* appeared to be similar in both control and test groups. Both populations contained some apparently normal fibroblasts but most showed lack of contact inhibition. In both cases there were also giant multinucleated and highly vacuolated cells, all of which persisted on subculture through several passages.

The electrophoretic mobilities of some of the cells rescued from the invasive primary tumours of the test group were found to be increased relative to the cells

FIG. 4.—Comparison of electrophoretic mobilities (EPM) of 33 cells rescued from 3 primary tumours derived from untreated BHK21 cells (control), and 56 cells rescued from 3 primary invasive tumours derived from histone treated BHK21 cells (test).

TABLE V.—*Malignancy of Cells Rescued from Primary Tumours Derived from Untreated and Histone Treated BHK21 Cells*

| | CLAC hamsters $5 \times 10^4$ cells | | | |
|---|---|---|---|---|
| Controls | Growth time (weeks) | Tumour weight (g) | Invasive site | Secondaries |
| 1 | 8 | 4·9 | none | none |
| 2 | 8 | 11·4 | none | none |
| 3 | 8 | 7·5 | none | none |
| Tests | | | | |
| 1 | 8 | 14·6 | Body wall | Whole viscera |
| 2 | 8 | 28·8 | Body wall | Whole viscera |
| 3 | 7 | 9·4 | Lymphatics | Lymph node and subcutaneous secondary |

from the non-invasive tumours of the control group, and 2 electrophoretically distinct populations of cells appeared to have evolved (Fig. 4). Further, after subculture 5 times *in vitro*, the rescued cells were found to maintain their respective tumour producing properties when re-inoculated into hamsters (Table V). Only those from the " histone " tumours gave rise to invasion and metastases.

### DISCUSSION

No statistically significant difference could be found in any of the measurements made on the primary tumours between the control and test groups. However, most of the tumours in the test group invaded either the epidermis or body wall whereas none in the control group invaded. Moreover, several of the test tumours were capable of causing extensive metastases. Thus, these tumours were behaving in a truly malignant fashion as compared with those in the control group which produced localized encapsulated growths. The finding that cells obtained from the invasive test tumours had a higher electrophoretic mobility than their corresponding controls correlates well with the observations made by Purdom, Ambrose and Klein (1958) on the progressive increase in negative electrical charge of cells with increasing malignancy. It is also significant that these invasive tumour cells, after 5 subcultures *in vitro*, maintained their increased malignancy on re-inoculation into hamsters. This must mean that it was highly unlikely that residual histone played any part.

Since the invasive tumours were derived from cells challenged *in vitro* with crude rat liver histone, it follows that the preparation affected cells in the population so as to increase their malignant potential. The exact nature of the histone–cell interaction is, however, difficult to assess although the evidence is in favour of a transforming rather than a selective process, since no evidence of histone toxicity was apparent.

The possibility exists that some cells in the BHK21 populations were potentially invasive, and that the crude histone challenge *in vitro* in some way encouraged their growth whilst suppressing that of the non-invasive cells. In this way, although the viability of the cell population taken as a whole may not apparently be altered, individual invasive cells could be given a selective advantage. It is difficult to imagine, however, how the crude histone preparation could selectively encourage the growth of malignant cells whilst at the same time inhibit the growth of non-invasive cells. In any event, if this hypothesis were correct one might have expected that a few of the postulated invasive cells in the control cultures would have produced invasive tumours in the control group of hamsters, a phenomenon which in fact was remarkably absent in our experiments.

It would seem therefore that a " transformation " mediated by some component or components in our crude rat liver histone is the only simple explanation which corresponds with all the data. It may be that previously untranscribed native DNA was becoming expressed on crude histone treatment or, conversely, that previously transcribed genes controlling growth characteristics were inactivated. It is perhaps important to emphasize here that our histone preparation produced very faint bands in addition to the characteristic histone fractions, on polyacrylamide gel electrophoresis (Panyim and Chalkley, 1969). We consider these extra bands to be polymers of fraction F3, but the possibility exists that they represent non-histone material which could be responsible for the results reported here.

Although the role of histones in the control of genetic expression is still not yet resolved, it would not seem unreasonable to postulate that the rat liver histone preparation added to the cells *in vitro* was in some way altering the phenotypic expression of these cells. There is certainly no lack of evidence that histones are taken up into cells (Levine *et al.*, 1968; Ryser and Hancock, 1965; Becker and Green, 1960), and the recent observations made in this laboratory concerning increases in nuclear size during histone challenge

(Latner, Longstaff and Lunn, unpublished observations), together with the altered cell activity reported here, suggest that the added histones were altering genetic expression and possibly allowing the transcription of a latent oncogene.

We are at present carrying out studies with purified histones and early results, especially in regard to nuclear size, seem to encourage the concept that histones alone could be the effective factor. We are also planning to extend our work to other non-metastasizing tumour systems, in order to discover whether the effect we have described is a general phenomenon. In this context, however, it has already been shown (Latner *et al.*, 1971) that a crude histone preparation increased the *in vitro* invasive properties of the cell line Detroit 98, which is derived from human sternal marrow cells.

The authors wish to thank Professor M. G. P. Stoker for supplying the BHK21/C13 cell line. This work has received financial support from the North of England Council of the Cancer Research Campaign. The histones used in this investigation were prepared by Dr J. M. Lunn. We acknowledge the skilled technical assistance of Mr C. Cornell with the tissue cultures, and Mrs R. Darke for assistance with the animals used in this report.

### REFERENCES

BECKER, F. F. & GREEN, H. (1960) The Effects of Protamines and Histones on the Nucleic Acids of Ascites Tumour Cells. *Expl Cell Res.*, **19**, 361.

BONNER, J., CHALKLEY, G. R., DAHMUS, M., FARNBROUGH, D., FUJIMURA, F., HUANG, R. C., HUBERMAN, J., JENSEN, R., MARUSHIGE, K., OHLENSBUSCH, H., OLIVERA, B. & WIDHOLM, J. (1968) In *Methods in Enzymology* XII, Part B. Ed. B. S. P. Colowick and N. O. Kaplan. New York: Academic Press, p. 3.

BURTON, K. (1956) A Study of the Conditions and Mechanisms of the Diphenylamine Reaction for the Colorimetric Estimation of Deoxyribonucleic Acids. *Biochem. J.*, **62**, 315.

CAMPBELL, R. C. (1967) *Statistics for Biologists.* London: Cambridge University Press.

DULBECCO, R. & VOGT, M. (1954) Plaque Formation and Isolation of Pure Lines with Poliomyelitis Viruses. *J. Exp. Med.*, **99**, 167.

FISCHER, H. & WAGNER, L. (1954) Die Wirkung Niedermolekularer (Basischer) Proteine auf Zellen und Organischen. *Naturwissenschaften*, **41**, 533.

GOODWIN, B. C. & SIZER, I. W. (1965) Histone Regulation of Lactic Dehydrogenase in Embryonic Chick Brain Tissue. *Science, N.Y.*, **148**, 242.

HIRSCH, J. G. (1958) Bacteriocidal Action of Histone. *J. Exp. Med.*, **108**, 925.

JOHNS, E. W. & CONNORS, T. A. (1970) Specific Toxicity of Histone Fraction F2C Against TLX5 Lymphoma Ascites Cells *in Vitro*. *Nature, Lond.*, **228**, 1201.

LATNER, A. L. & LONGSTAFF, E. (1969) Modification by Crude Histones of Gene Activity for Lactate Dehydrogenase. *Nature, Lond.*, **224**, 71.

LATNER, A. L. & LONGSTAFF, E. (1971) Transformation of Mammalian Cells by Crude Histones. *Br. J. Cancer*, **25**, 280.

LATNER, A. L., LONGSTAFF, E. & LUNN, J. M. (1971) Invasive Properties of Histone Transformed Cells. *Br. J. Cancer*, **25**, 568.

LEVINE, A. S., NESBIT, M. E., WHITE, J. G. & YARBRO, J. W. (1968) Effects of Fractionated Histones on Nucleic Acid Synthesis in 6C3HED Mouse Ascites Tumour Cells and in Normal Spleen Cells. *Cancer Res.*, **28**, 831.

PANYIM, S. & CHALKLEY, R. (1969) The Heterogeneity of Histones I. A Quantitative Analysis of Calf Histones in Very Long Polyacrylamide Gels. *Biochemistry, N.Y.*, **8**, 3972.

PURDOM, L., AMBROSE, E. J. & KLEIN, G. (1958) A Correlation Between Electrical Surface Charge and Some Biological Characteristics During the Stepwise Progression of a Mouse Sarcoma. *Nature, Lond.*, **181**, 1586.

RYSER, H. J. P. & HANCOCK, R. (1965) Histones and Basic Polyamino Acids Stimulate the Uptake of Albumin by Tumour Cells in Culture. *Science, N.Y.*, **150**, 501.

VOROBYEV, V. I. & BRESLER, V. M. (1963) Unfractionated Preparations of Histones from Normal Mammalian Tissues as Agents Inhibiting Growth of Transplanting Tumours. *Nature, Lond.*, **198**, 545.